# HBV DNA level could predict significant liver fibrosis in HBeAg negative chronic hepatitis B patients with biopsy indication

**DOI:** 10.1186/s12876-014-0218-6

**Published:** 2014-12-19

**Authors:** Surat Praneenararat, Naichaya Chamroonkul, Pimsiri Sripongpun, Samornmas Kanngurn, Roongrueng Jarumanokul, Teerha Piratvisuth

**Affiliations:** Division of Gastroenterology, Department of Medicine, Prince of Songkla University, Songkhla, Thailand; Division of Pathology, Department of Pathology, Prince of Songkla University, Songkhla, Thailand; Division of Immunology, Prince of Songkla University, Songkhla, Thailand; NKC Institute of Gastroenterology and Hepatology, Prince of Songkla University, Songkhla, Thailand

**Keywords:** Predictor, Chronic hepatits B, HBeAg negative, HBV DNA, Liver fibrosis

## Abstract

**Background:**

Non-invasive models and methods to substitute liver biopsy in chronic hepatitis B (CHB) patients were investigated but their roles as predictors of significant liver histology for diagnosis of HBeAg-negative CHB patients who had indication for liver biopsy according to The American Association for the Study of Liver Diseases (AASLD) and The Asian Pacific Association for the Study of the Liver (APASL) guidelines are still unknown. This study was designed to identify predictors of significant liver necroinflammation as defined by a Histology Activity Index of necroinflammatory score ≥ 4 or Metavir necroinflammatory activity score ≥ 2 and significant liver fibrosis as defined by a Metavir fibrosis score ≥ 2 in HBeAg-negative CHB patients that had a hepatitis B virus (HBV) DNA level ≥ 2,000 IU/ml and age ≥ 40 years or elevated alanine aminotransferase level between 1–2 times the upper limit of normal.

**Methods:**

Twenty-two patients were prospectively included and performed liver biopsies. Clinical and laboratory parameters including age, gender, underlying disease, family history of cirrhosis or hepatocellular carcinoma, body mass index (BMI), HBV DNA level, HBsAg level, liver function test, complete blood count, aspartate aminotransferase-to-platelet ratio index and transient elastography were collected and analyzed with liver histology profiles.

**Results:**

Five patients (23%) had significant liver inflammation and 7 patients (32%) had significant liver fibrosis. Factors associated with significant liver inflammation were a lower BMI and higher alkaline phosphatase level while a factor associated with significant liver fibrosis was lower age. On multivariate analysis, only HBV DNA level > 5.5 log IU/ml could predict significant liver fibrosis (odds ratio 28.012, 95% CI, 1.631-481.240, *p* = 0.022) and its sensitivity, specificity, positive predictive value and negative predictive value were 71.4%, 93.3%, 83.3% and 87.5% respectively.

**Conclusions:**

An HBV DNA level of > 5.5 log IU/ml was able to predict significant liver fibrosis for treatment of HBeAg-negative CHB patients that had indication for liver biopsy as recommended by AASLD and APASL guidelines.

## Background

Liver inflammation and fibrosis evaluations are important elements of a severity assessment for treatment decisions in the management of chronic hepatitis B (CHB) patients. The gold standard for evaluation in grading of liver inflammation and staging of liver fibrosis is a liver biopsy [1–3]. However, biopsy is an invasive procedure with minimal but significant risk of morbidity and mortality [4].

Non-invasive models and methods including transient elastography (TE) and aspartate aminotransferase-to-platelet ratio index (APRI) have been investigated and shown to predict liver histology in CHB patients with fair accuracy [5]. However, their accuracy in liver histology prediction of the HBeAg-negative CHB patient subgroup, who would require a liver biopsy as The American Association for the Study of Liver Diseases (AASLD) and The Asian Pacific Association for the Study of the Liver (APASL) guidelines recommend, is still unknown.

The aim of the present study was to find, through a prospective cross-sectional study, the clinical and laboratory parameters that could predict liver histology in HBeAg-negative CHB patients who required evaluation of liver inflammation and fibrosis by liver biopsy in accordance with AASLD and APASL guidelines which are a hepatitis B virus (HBV) DNA level ≥ 2,000 IU/ml and age ≥ 40 years, or an elevated alanine aminotransferase (ALT) level between 1–2 times the upper limit of normal (ULN) [1,3]. If a predictor is found, it might become an alternative method for liver inflammation and fibrosis assessment in this subgroup of patients.

## Methods

### Patients

All CHB patients who had visited the outpatient clinic of the Division of Gastroenterology or the NKC institute of Gastroenterology and Hepatology, Prince of Songkla University, Songkhla, Thailand between November 2012 and February 2014 were screened for inclusion in the study.

Inclusion criteria were age of more than 20 years, positive HBsAg for more than 6 months, negative HBeAg, HBV DNA ≥ 2,000 IU/ml, no previous HBV treatment and positive indication for liver biopsy according to AASLD and APASL guidelines [1,3] including ALT 40–79 U/L (1–2 times of ULN) or age ≥ 40 years. The authors also added positive family history of cirrhosis or hepatocellular carcinoma (HCC) as an additional indication for liver biopsy due to Thai national guidelines [6] (only 1 patient biopsied by this indication was included).

Exclusion criteria were co-infection with hepatitis C virus or human immunodeficiency virus infection, alcohol intake of more than 20 g/day, cirrhosis as documented by either physical examination, laboratory or radiology findings, HCC, pregnancy, contraindication to liver biopsy including the presence of ascites, an international normalized ratio over 1.5, activated partial thromboplastin time > 1.5 times or platelets < 100,000/ml [4] and refusal to participate in the study. Written informed consent was obtained from all patients before enrollment. The study was approved by the Ethical Committee of Prince of Songkla University and in accordance with the Helsinki Declaration of 1975.

### End points

The primary end point was the discovery of the factors that may predict liver histology in HBeAg-negative CHB patients that needed liver biopsy according to AASLD and APASL guidelines. The secondary end points were the associations between clinical and laboratory parameters with liver histology.

### Study design

Clinical and laboratory data for all of the eligible patients were collected. They underwent liver stiffness (LS) measurement by TE within 2 weeks of the day of liver biopsy. The clinical and laboratory data gathered for the participants were age, gender, underlying disease, family history of cirrhosis or HCC, body mass index (BMI), HBsAg level, HBV DNA level, liver function test, complete blood count, α-fetoprotein (AFP) and APRI.

### Quantification of HBsAg and HBV DNA Levels

Serum HBsAg levels were quantified using the Architect HBsAg QT (Abbott Laboratories) according to manufacturer’s instructions [7,8] with a detection range of 0.05 to 250 IU/ml. If the HBsAg level was more than 250 IU/ml, samples were diluted to 1:100 to 1:1000 to obtain a reading within the calibration curve range. Serum HBV-DNA levels were quantified with a COBAS AmpliPrep/COBAS TaqMan assay (Roche Diagnostic Systems Inc, Mannheim, Germany) with a detection range of 20–1.7×10^8^ IU/ml.

### LS measurements by TE

LS was determined using Fibroscan® (Echosens, Paris, France) as described by Sandrin [9]. The TE operator was a physician who had previously performed LS by Fibroscan® on at least 50 patients (N. J.). The operators performed at least 10 valid measurements at each time. LS values are expressed in median (kilopascal; kPa). LS values included in the study needed an interquartile range of less than 30% over the median ratio and a success rate of more than 80%.

### Liver histology

Liver histology was evaluated by a pathologist (S.K.) expert in gastroenterological pathology, blinded to any other clinical and laboratory data and tested for intra-observer variation. Liver samples needed to be at least 2 cm in length and have at least 11 complete portal tracts for histological evaluation [10]. The degrees of liver necroinflammation were assessed against the Histology Activity Index (HAI) necroinflammatory activity score (from 0–18) [11] and the Metavir necroinflammatory activity score (from 0–4). The degrees of liver fibrosis were assessed using the Metavir fibrosis score (range 0–4) [12].

### Definition

Significant liver inflammation was defined as a HAI necroinflammatory activity score of ≥ 4 or Metavir necroinflammatory activity score of ≥ 2, while significant liver fibrosis was defined by a Metavir fibrosis score of ≥ 2. If one of the above definitions were satisfied, they could be referred to as significant liver histology. These levels represented at least moderate inflammation and significant fibrosis and influenced the management of the patients in terms of treatment indications.

### Statistical analysis

The baseline characteristics were presented as means with standard deviation. Comparison of means used an Independent-samples t test or Mann–Whitney U test according to data distributions. Factors that had *p-*value < 0.1 were further subjected to univariate analysis with a Fisher exact test and multivariate analysis by multiple logistic regressions. Statistical significance was set at *p*-value < 0.05.

## Results

### Patient characteristics

Twenty-two eligible patients were included in the study. Baseline characteristics of all patients divided according to liver pathology are shown in Table 1. The number of males and females was equal, as well as positive or negative family history of cirrhosis or HCC. The mean age of participants was 48 years. There were 4 (18%) patients with both significant liver inflammation and fibrosis, 1 (5%) patient with only significant inflammation and 3 (14%) patients with significant liver fibrosis only. The indication for liver biopsy in 4 patients under 40 years of age were elevated ALT in 3 patients and a positive family history of cirrhosis or HCC in 1 patient. The pathological finding in this group resulted in significant liver fibrosis in 3 patients and both significant liver inflammation and fibrosis in 1 patient.

**Table 1:** **Baseline characteristics of 22 HBeAg-negative CHB patients included in the study**

**Parameters**	**All patients (N = 22)**	**Min - Max**	**Liver inflammation**			**Liver fibrosis**			**Liver inflammation or fibrosis**		
			**Significant** ^**a**^ **(n = 5)**	**Insignificant (n = 17)**	***p*** **-value**	**Significant** ^**b**^ **(n = 7)**	**Insignificant (n = 15)**	***p*** **-value**	**Significant** ^**c**^ **(n = 8)**	**Insignificant (n = 14)**	***p*** **-value**
Male/female, n (%)	11/11(50/50)	-	1/4(5/18)	10/7(45/32)	0.127	4/3(18/14)	7/8(32/36)	0.647	4/7(18/32)	4/7(18/32)	1.000
Age, mean ± SD, years	48 ± 9	35 - 65	41.8 ± 8.3	50.1 ± 8.4	0.068	42.7 ± 8.4	50.7 ± 8.2	0.047	44.4 ± 9.1	50.4 ± 8.4	0.134
BMI, mean ± SD, kg/m^2^	24.54 ± 3.76	20.28 – 35.75	22.09 ± 1.51	25.26 ± 3.94	0.046	22.91 ± 1.64	25.30 ± 4.25	0.217	22.73 ± 1.61	25.57 ± 4.27	0.088
Positive family history of cirrhosis or HCC, n (%)	11/11(50)	-	3/2(14/9)	8/9(36/41)	0.611	3/4(14/18)	8/7(36/32)	0.647	4/7(18/32)	4/7(18/32)	1.000
HBsAg levels, mean ± SD, log IU/mL	3.36 ± 0.55	1.57 – 4.24	3.01 ± 0.84	3.46 ± 0.42	0.225	3.39 ± 0.24	3.34 ± 0.66	0.698	3.16 ± 0.68	3.47 ± 0.46	0.275
HBV DNA levels, mean ± SD, log IU/mL	4.92 ± 1.21	3.59 – 7.56	5.45 ± 1.55	4.76 ± 1.09	0.327	5.63 ± 1.30	4.59 ± 1.04	0.053	5.40 ± 1.38	4.65 ± 1.05	0.172
TB, mean ± SD, mg/dL	0.55 ± 0.22	0.17 – 0.96	0.40 ± 0.11	0.60 ± 0.23	0.08	0.44 ± 0.20	0.61 ± 0.22	0.109	0.46 ± 0.19	0.61 ± 0.23	0.120
DB, mean ± SD, mg/dL	0.15 ± 0.07	0.03 – 0.29	0.13 ± 0.05	0.16 ± 0.07	0.288	0.14 ± 0.08	0.16 ± 0.06	0.458	0.14 ± 0.07	0.16 ± 0.07	0.580
AST, mean ± SD, mg/dL	30.3 ± 11.4	15 - 64	41.0 ± 16.8	27.1 ± 7.3	0.071	34.0 ± 15.8	28.5 ± 8.8	0.572	35.5 ± 15.2	27.3 ± 7.6	0.231
ALT, mean ± SD, mg/dL	35.4 ± 17.3	13 - 70	47.4 ± 21.8	31.9 ± 1.47	0.077	36.1 ± 20.1	35.1 ± 1.64	0.896	38.8 ± 20.4	33.5 ± 15.8	0.507
ALP, mean ± SD, mg/dL	65.2 ± 16.7	40 - 109	85.0 ± 16.7	59.4 ± 11.7	0.001	69.3 ± 16.7	63.3 ± 16.9	0.443	74.3 ± 20.9	60.0 ± 11.6	0.051
GGT, mean ± SD, mg/dL	22.3 ± 12.5	9 - 63	24.8 ± 10.6	21.5 ± 13.2	0.347	23.9 ± 10.7	21.5 ± 13.5	0.459	24.1 ± 9.9	21.2 ± 14.0	0.274
Albumin, mean ± SD, g/dL	4.47 ± 0.13	4.2 – 4.7	4.40 ± 0.16	4.49 ± 0.11	0.153	4.44 ± 0.17	4.49 ± 0.11	0.468	4.46 ± 0.17	4.48 ± 0.11	0.784
Globulin, mean ± SD, g/dL	2.96 ± 0.34	2.20 – 3.50	3.16 ± 0.27	2.91 ± 0.35	0.151	3.03 ± 0.36	2.93 ± 0.35	0.559	3.00 ± 0.34	2.94 ± 0.36	0.718
INR, mean ± SD,	0.98 ± 0.07	0.88 – 1.14	1.00 ± 0.11	0.97 ± 0.05	0.527	0.99 ± 0.07	0.97 ± 0.07	0.554	1.01 ± 0.88	0.96 ± 0.05	0.103
White blood cells, mean ± SD, /mm^3^	6,650 ± 1,329	4,880 – 9,100	7,168 ± 1,267	6,497 ± 1,345	0.333	6,803 ± 1,209	6,578 ± 1,416	0.721	6,773 ± 1,123	6,579 ± 1,470	0.752
Absolute neutrophil count, mean ± SD, /mm^3^	3,456 ± 1,132	1,819 – 6,825	4,302 ± 1,691	3,207 ± 823	0.055	3,762 ± 1,608	3,313 ± 863	0.399	3,748 ± 1,489	3,289 ± 890	0.372
Absolute lymphocyte count, mean ± SD /mm^3^	2,405 ± 565	1,249 – 3,583	2,222 ± 527	2,459 ± 579	0.423	2,339 ± 531	2,435 ± 595	0.719	2,345 ± 492	2,439 ± 618	0.718
Hemoglobin, mean ± SD, g/dL	13.5 ± 1.3	9.8 – 15.6	13.7 ± 1.1	13.5 ± 1.4	0.781	13.6 ± 1.0	13.5 ± 1.5	0.793	13.8 ± 1.0	13.4 ± 1.5	0.503
Platelet, mean ± SD, x10^3^/mm^3^	234.5 ± 50.1	143 - 327	223.4 ± 70.2	237.8 ± 44.8	0.586	215.3 ± 55.7	243.5 ± 46.5	0.228	222.6 ± 55.6	241.2 ± 47.5	0.414
AFP, mean ± SD, ng/mL	2.71 ± 1.47	0.99 – 6.40	1.92 ± 0.62	2.95 ± 1.57	0.256	2.04 ± 0.73	3.03 ± 1.63	0.217	2.07 ± 0.68	3.08 ± 1.68	0.246
LS values, mean ± SD, kPa	7.12 ± 2.92	2.50 – 12.30	9.16 ± 3.00	6.59 ± 2.72	0.084	8.06 ± 2.56	6.77 ± 3.06	0.347	8.59 ± 2.83	6.37 ± 2.75	0.087
APRI, mean ± SD	0.35 ± 0.21	0.15 – 1.12	0.54 ± 0.37	0.30 ± 0.09	0.210	0.45 ± 0.33	0.30 ± 0.10	0.283	0.45 ± 0.31	0.30 ± 0.10	0.206

*Abbreviations:* *CHB* chronic hepatitis B, *BMI* body mass index, *TB* total bilirubin, *DB* direct bilirubin, *AST* aspartate aminotransferace, *ALT* alanine aminotransferase, *ALP* alkaline phosphatase, *GGT* gamma glutamyltransferase, *INR* international normalized ratio, *AFP* α-fetoprotein, *LS* liver stiffness, *APRI* AST to platelet ratio index.

^a^Significant liver inflammation (HAI necroinflammatory activity score ≥4 or Metavir necroinflammatory activity score ≥2).

^b^Significant liver fibrosis (Metavir fibrosis score ≥2).

^c^Significant liver inflammation or fibrosis.

The patients with significant liver inflammation had a significantly lower BMI (22.09 ± 1.51 kg/m^2^ Vs 25.26 ± 3.94 kg/m^2^, *p* = 0.046) and higher alkaline phosphatase (85.0 ± 16.7 mg/dL Vs 59.4 ± 11.7, *p* = 0.001) than the patients without significant liver inflammation while patients with significant liver fibrosis were significantly younger (42.7 ± 8.4 years Vs 50.7 ± 8.2, *p* = 0.047) than those without significant liver fibrosis. When combined into the same group, the patients with significant liver inflammation and significant liver fibrosis showed no significant differences in baseline characteristics compared with patients without significant liver histology.

### Predictors of significant liver histology in HBeAg-negative CHB patients

Many factors were found to predict significant liver inflammation in univariate analysis, including age ≤ 42 years, BMI < 24.5 kg/m^2^, total bilirubin < 0.45 mg/dl, aspartate aminotransferace > 30 mg/dl, alkaline phosphatase > 78 mg/dl, absolute neutrophil count > 4,300/mm^3^, ceruloplasmin > 20 mg/dl and LS values of ≥ 11.5 kPa (Table 2). Nevertheless, these factors didn’t reach statistical significance in multivariate analysis (data not shown). The same results were also discovered in the group with significant liver inflammation or fibrosis: a BMI of < 25 kg/m and ALP > 78 mg/dl were associated with significant liver histology in univariate analysis, but they were not found to be significant in the multivariate analysis (data not shown).

**Table 2:** **Univariate analysis in prediction of significant liver histology**

**Parameters**	**Significant liver inflammation**			**Significant liver fibrosis**			**Significant liver inflammation or fibrosis**		
	**n/N**	**Percent**	***p-*** **value**	**n/N**	**Percent**	***p-*** **value**	**n/N**	**Percent**	***p-*** **value**
Age ≤ 40 years	3/7	42.9	0.160	4/7	57.1	0.107			
Age ≤ 42 years	4/8	50	0.039	5/8	62.5	0.032			
Age ≤ 50 years	4/11	36.4	0.155	5/11	45.5	0.181			
BMI < 23 kg/m^2^	4/9	44.4	0.067				5/9	55.6	0.135
BMI < 24.5 kg/m^2^	5/13	38.5	0.049				7/13	53.8	0.052
BMI < 25 kg/m^2^	5/15	33.3	0.114				8/15	53.3	0.020
TB < 0.45 mg/dL	4/8	50	0.039						
AST > 19 mg/dL	4/19	21.1	0.558						
AST > 30 mg/dL	4/8	50	0.021						
AST > 40 mg/dL	3/5	60	0.055						
AST > 50 mg/dL	1/1	100	0.227						
ALT > 30 mg/dL	4/12	33.3	0.218						
ALT > 40 mg/dL	3/8	37.5	0.233						
ALP > 78 mg/dL	4/5	80	0.003				4/5	80	0.039
Absolute neutrophil count > 4,300/mm^3^	3/4	75	0.024						
LS values ≥ 7.0 kPa	3/10	30	0.406				5/10	50	0.221
LS values ≥ 8.5 kPa	3/5	60	0.233				5/8	62.5	0.072
LS values ≥ 9.5 kPa	3/5	60	0.055				3/5	60	0.233
LS values ≥ 11.5 kPa	2/2	100	0.043				2/2	100	0.121
HBV DNA levels > 4 log IU/mL				5/14	35.7	0.490			
HBV DNA levels > 5 log IU/mL				5/11	45.5	0.181			
HBV DNA levels > 5.5 log IU/mL				5/6	83.3	0.004			
HBV DNA levels > 6 log IU/mL				3/4	75	0.077			

In the group with liver fibrosis, age ≤ 42 years and HBV DNA levels > 5.5 log IU/ml were predictors of significant liver fibrosis in univariate analysis as a predictor of significant liver fibrosis in univariate analysis. HBV DNA levels > 5.5 log IU/ml was the only factor that was found as a significant predictor from multivariate analysis [odds ratio (OR) 28.012, 95% CI, 1.631-481.240*, p* = 0.022] (Table 3). Sensitivity, specificity, positive predictive value and negative predictive value were 71.4%, 93.3%, 83.3% and 87.5% respectively.

**Table 3:** **Multivariate analysis in prediction of significant liver fibrosis**

**Parameters**	**Odds ratio**	***p-*** **value**	**95% confidence interval**
Age ≤ 42 years	7.403	0.139	0.523-104.716
HBV DNA levels > 5.5 log IU/mL	28.012	0.022	1.631-481.240

## Discussion

In management of CHB patients, histological liver evaluation is an important part of the severity assessment and has importance for treatment decisions. Although liver biopsy is a gold standard to determine the stage of fibrosis and degree of necroinflammation, it is an invasive procedure with minimal but significant risk of morbidity and mortality [4]. Therefore, many non-invasive methods have been applied to replace this invasive procedure.

With respect to liver fibrosis, the HBV DNA level was the only factor that reached statistical significance with an OR of 28.012 at a level of over 5.5 log IU/ml. This finding is supported by other studies. For example, in the study by Croagh et al. [13] HBV DNA level was found to be a predictor of significant fibrosis in HBeAg-negative CHB patients with varying ALT with an OR of 1.3 for every 1 log increment [13]. Association was also found in the study by Mohamadnejad et al. [14] with the sensitivity of 74% and specificity of 80% at cutoff level to 4.91 log IU/ml [14]. HBV DNA levels also correlated with advanced fibrosis in HBeAg-negative CHB patients with normal ALT and varying ages in Xiao et al. [15]. In contrast, this finding was not found in HBeAg-positive CHB patients [13–16]. Further studies are necessary to explain variable outcomes between different phases of CHB patients. For this reason, HBV DNA level with a cut-off value at 5.5 log IU/ml might be a promising serum marker in predicting liver fibrosis in treatment naïve HBeAg-negative CHB patients with positive indication for liver biopsy as recommended by AASLD and APASL guidelines.

Younger age was another factor found to be associated with liver fibrosis. This was against the basic knowledge that fibrosis would progress and eventually develop into cirrhosis with more advanced age [1–3]. This finding might be explained by selection bias. The authors excluded cirrhosis which could be a final outcome in younger patients with significant histology, though patients of more advanced age but without cirrhosis might represent a group of patients which actually had an insignificant histology. If cirrhosis was included in the present study, the result should have been opposite.

Although LS values and APRI correlated with degree of liver fibrosis, it was not associated with significant liver fibrosis in the present study. LS values seemed to accurately predict advanced fibrosis especially stage 4 fibrosis or cirrhosis from meta-analysis studies, but unfortunately, its accuracy as a predictor of significant liver fibrosis (fibrosis stage ≥ 2) was just fair. These studies demonstrate that LS values between fibrosis stage 1 and 2 were similar [17–19]. LS values might be a tool to predict advanced but not significant fibrosis, thus it should not be used to guide treatment initiation decisions except where LS values are high enough to reflect advanced fibrosis or cirrhosis. Not surprisingly, APRI was not associated with significant liver histology. This correlated with the study by Degos et al. [20] where APRI also had better accuracy in diagnosis of cirrhosis than significant fibrosis [20]. In contrast, higher LS values tended to be associated with significant liver inflammation. Moreover, LS values ≥ 11.5 kPa also predicted significant liver inflammation in univariate analysis. These findings are supported by previous knowledge that hepatic inflammation is one of the confounding factors in LS values [21,22]. Thus, LS values as found in the patient group in this study might really represent liver inflammation, not liver fibrosis.

Patients with significant liver inflammation had significantly lower BMI and higher alkaline phosphatase than another groups. However, these findings were not found in other studies in HBeAg-negative CHB patients with varying degrees of ALT [13–15]. Larger trials in the same fashion as the present study are needed to clarify the influence of these factors.

Nowadays, serum HBsAg level is considered to be a useful test that can be used in many conditions in CHB patients. It could predict HBV levels and liver-related diseases, and also significant liver fibrosis in HBeAg-positive CHB patients [23–26]. While it was also included in the present study, it was not found to be associated with any significant histology. Martinot-Peignoux et al. [27] also found the same results with HBeAg-negative CHB patients with varying degrees of ALT [27]. These findings suggest that serum HBsAg level is not a potential tool in liver histology assessment in HBeAg-negative CHB patients.

There is no predictor for significant liver histology in the current study. Even if HBV DNA level was found to predict significant liver fibrosis, it was not associated with significant liver histology. An insignificant association with significant liver inflammation and the small number of patients with significant liver histology are possible reasons to explain this finding.

The strength of the present study is the inclusion criteria used. The authors gathered patients in a prospective manner and strictly selected those that really needed liver biopsy as recommended in guidelines, though the result could also be applied in clinical practice. Thus, the small number of total patients is a limitation in the present study.

## Conclusions

A serum HBV DNA level of > 5.5 log IU/ml was found to predict significant liver fibrosis in treatment naïve HBeAg-negative CHB patients that had indication for liver biopsy as recommended by AASLD and APASL guidelines.

## Abbreviations

AASLD: The American Association for the Study of Liver Diseases
AFP: α-fetoprotein
ALT: Alanine aminotransferase
APASL: The Asian Pacific Association for the Study of the Liver
APRI: Aspartate aminotransferase-to-platelet ratio index
BMI: Body mass index
CHB: Chronic hepatitis B
HAI: Histology Activity Index
HBV: Hepatitis B virus
HCC: Hepatocellular carcinoma
kPa: kilopascal
LS: Liver stiffness
OR: Odd ratio
TE: Transient elastography
ULN: Upper limit of normal
